# Inconsistent radiographic diagnostic criteria for lisfranc injuries: a systematic review

**DOI:** 10.1186/s12891-023-07043-z

**Published:** 2023-11-27

**Authors:** Dexter Seow, Youichi Yasui, Li Yi Tammy Chan, Gareth Murray, Maya Kubo, Masashi Nei, Kentaro Matsui, Hirotaka Kawano, Wataru Miyamoto

**Affiliations:** 1 National University Health System, Singapore, Singapore; 2 Yong Loo Lin School of Medicine, National University of Singapore, Singapore, Singapore; 3 Department of Orthopaedic Surgery, School of Medicine, Teikyo University, 2-11-1, Kaga, Itabashi, Tokyo, 173-8605 Japan; 4 Royal College of Surgeons in Ireland, Dublin, Ireland

**Keywords:** Diagnostic imaging, Midfoot, Tarsometatarsal, Trauma, X-ray

## Abstract

**Purpose:**

To evaluate the radiographic diagnostic criteria and propose standardised radiographic criteria for Lisfranc injuries.

**Methods:**

A systematic review of the PubMed and Embase databases was performed according to the PRISMA guidelines. The various radiographic criteria for the diagnosis of Lisfranc injuries were extracted. Descriptive statistics were presented for all continuous (as mean ± standard deviation) and categorical variables (as frequencies by percentages).

**Results:**

The literature search included 29 studies that totalled 1115 Lisfranc injuries. The risk of bias ranged from “Low” to “Moderate” risk according to the ROBINS-I tool. The overall recommendations according to the GRADE assessment ranged from “Very Low” to “High”. 1^st^ metatarsal to 2^nd^ metatarsal diastasis was the most common of the 12 various radiographic diagnostic criteria observed, as was employed in 18 studies. This was followed by 2^nd^ cuneiform to 2^nd^ metatarsal subluxation, as was employed in 11 studies.

**Conclusion:**

The radiographic diagnostic criteria of Lisfranc injuries were heterogeneous. The proposition for homogenous radiographic diagnostic criteria is that the following features must be observed for the diagnosis of Lisfranc injuries: 1^st^ metatarsal to 2^nd^ metatarsal diastasis of ≥ 2 mm on anteroposterior view or 2^nd^ cuneiform to 2^nd^ metatarsal subluxation on anteroposterior or oblique views. Further advanced imaging by CT or MRI may be required in patients with normal radiographs but with continued suspicion for Lisfranc injuries.

**Level of evidence:**

4, systematic review.

## Introduction

Lisfranc injury is a midfoot injury that refers to the displacement of one or more of the metatarsi from the tarsus [1]. The incidence is low, with approximately 0.2% of all fractures affecting one in every 55,000 people in the United States [2]. The wide-ranging characteristics of Lisfranc injury have been well documented, from low-energy ligamentous injuries commonly associated with sports activities to high-energy crushing injuries in traumatic events [1]. Systematic reviews have indicated that reasonable clinical outcomes can be expected in patients despite the wide-ranging characteristics of Lisfranc injury [3–6]. However, the diagnosis of Lisfranc injuries has remained a challenge, and is estimated to have been commonly missed or misdiagnosed in 20% to 24% of cases [7, 8]. Therefore, the current literature has recommended that clinicians obtain radiological imaging in patients with a highly suspicious history and/or physical findings of Lisfranc injuries [1].

A variety of radiological imaging modalities, that are radiographs, computed tomography (CT), and magnetic resonance imaging (MRI), can diagnose Lisfranc injuries [1, 9]. Among these varieties, radiographs remain the first line for demonstrating structural bony and soft tissue abnormalities, as image acquisition is non-invasive, inexpensive, and rapidly available [10]. However, the radiographic criteria for diagnosing Lisfranc injuries have been variable [11]. The establishment of standardised radiographic criteria for Lisfranc injuries can add foresight to the clinical decision-making of treatment choice and subsequently enhance patient consultation.

Therefore, the purpose of this study is to evaluate the radiographic diagnostic criteria and propose standardised radiographic criteria for Lisfranc injuries. The hypothesis is that the diagnostic criteria of Lisfranc injuries are heterogenous in the current literature.

## Methods

### Study design, search strategy and study identification

A systematic review of the PubMed and Embase databases was performed by two authors (D.S. and L.Y.T.C.) using specific search terms and eligibility criteria according to the Preferred Reporting Items for Systematic Reviews and Meta-Analyses (PRISMA) guidelines from inception to April 11, 2022 [12]. The purpose of this systematic review was to evaluate patients with Lisfranc injuries (P) diagnosed using radiological imaging and criteria (O) in clinical cohort studies, whereby P is the population, and O is the outcome based on the PICO framework of clinical research questions. The search terms were: (Lisfranc OR tarsometatarsal OR midfoot) AND (injury OR injuries OR fracture OR fractures OR dislocation OR dislocations OR displacement OR diastasis OR subluxation OR rupture OR ruptures OR sprain). The titles, abstracts and full texts were screened using specific eligibility criteria. The references of full-text studies for review were additionally screened for studies unidentified by the search strategy. Studies were included by the agreement of both authors and differences resolved by the senior author (Y.Y.).

### Eligibility criteria

The inclusion criteria were: 1) clinical studies that used radiographic criteria for identification of Lisfranc injuries, 2) full-text studies and 3) written in English. The exclusion criteria were: 1) animal studies, 2) cadaver studies, 3) case reports, 4) in vitro studies and 5) reviews.

### Assessment of evidence

The level of evidence (LoE) was assessed using the criteria by *The Journal of Bone & Joint Surgery* [13]. The above criteria use a hierarchical rating to evaluate the LoE from Level I through V based on the study design used to answer the primary research question. Level I studies has the highest QoE (randomised controlled trials), followed sequentially by Level II (prospective cohort), III (retrospective cohort), IV (case series), and V (non-clinical studies, case reports). The risk of bias (RoB) was assessed using the Risk of Bias in Non-Randomised Studies of Interventions (ROBINS-I) tool. The ROBINS-I tool rates studies as “Low”, “Moderate”, “Serious”, and “Critical” risk of bias based on the domains: bias due to confounding, selection of participants, classification of interventions, deviations from intended interventions, missing data, measurement of outcomes, and selection of the reported results [14]. The quality of evidence was evaluated using the Grades of Recommendation, Assessment, Development and Evaluation (GRADE) approach [15].

### Data extraction and categorization

Data was extracted onto a Microsoft® Excel datasheet version 16 (Microsoft® Excel for Mac, Redmond, WA). The study/patient characteristics extracted were: Lisfranc injuries (n), sex, mean age and follow-up. The radiographic diagnostic characteristics extracted were: weightbearing condition(s) and if the contralateral radiograph was obtained. Radiographic criteria pertaining to the diagnosis of Lisfranc injuries were then extracted, with these consistencies evaluated across all the included studies.

Statistical analysis was performed using R version 3.5.1 (R Foundation for Statistical Computing, Vienna, Austria). Descriptive statistics were presented for all continuous and categorical variables. Continuous variables were presented as mean ± standard deviation and categorical variables as frequencies by percentages. A value of *p* < 0.05 was considered statistically significant.

## Results

### Literature search and study/patient characteristics (Table 1)

**Table 1:** Study/patient characteristics

Study	LoE	Lisfranc injuries (n)	Gender (male; female)	Mean age, years (range)	Follow-up, months (range)
Faciszewski et al. *J Bone Joint Surg Am.* 1990. [16]	4	15	10; 5	38.7 (19 to 75)	24 to 156
Curtis et al. *Am J Sports Med.* 1993. [17]	4	19	14; 5	25.5 (17 to 42)	25 (15 to 45)^a^
Shapiro et al. *Am J Sports Med.* 1994. [18]	4	9	5; 4	23.7 (18 to 45)	34.1 (12 to 52)^a^
Kinik et al. *Foot Ankle Surg.* 1999. [19]	4	11	8; 3	31.2 (16 to 44)	40.8^a^
Nunley and Vertullo. *Am J Sports Med.* 2002. [20]	3	15	13; 2	21 (15 to 32)	27 (9 to 72)^a^
Perugia et al. *Int Orthop.* 2003. [21]	4	42	28; 14	37.7 (17 to 70)	58.4 (24 to 84)^a^
Ly and Coetzee. *J Bone Joint Surg Am.* 2006. [22]	1	41	NR	32.4 (19 to 52)	42.5 (25 to 60)^a^
Reinhardt et al. *Foot Ankle Int.* 2012. [23]	3	25	8; 17	46 (20 to 73)^b^	42 (24 to 96)^a^
Crates et al. *J Foot Ankle Surg.* 2015. [24]	3	36	18; 18	Sx, 29.6 (16 to 57); Cx, 36.7 (15 to 63)^a^	Sx, 33 (12 to 60); Cx, 36 (12 to 59)^a^
Miyamoto et al. *Arch Orthop Trauma Surg.* 2015. [25]	4	5	4; 1	19.4 (17 to 21)	18.8 (12 to 26)^a^
Cassinelli et al. *Foot Ankle Int.* 2016. [26]	4	8	1; 7	39.8 (18 to 60)	37.2 (24 to 69.6)^a^
Del Vecchio et al. *Adv Orthop.* 2016. [27]	4	5	1; 4	42.4 (25 to 67)	19.4 (18 to 21)^a^
Lien et al. *J Foot Ankle Surg.* 2017. [28]	4	10	7; 3	35.2 (19 to 72)	6^c^
Seo et al. *Foot Ankle Int.* 2017. [29]	3	51	28; 23	34.5 (NR)	NR
Gee et al. *Curr Orthop Pract.* 2019. [30]	3	12	10; 2	SF, 25.7 (NR); SB, 29.7 (NR)	12.3 (5.6 to 30.0)^a^
Pigott et al. *Foot Ankle Spec.* 2019. [31]	3	45	22; 23	35.8 (19 to 60)	31.4 (6 to 119)^a^
Porter et al. *Foot Ankle Int.* 2019. [32]	4	82	64; 18	21.0 (12 to 40)	TD, > 12; MCD, > 12; PED, 6 to 12^c^
Ren et al. *Chin J Traumatol.* 2019. [33]	3	61	38; 23	39.4 (19 to 64)	12.3 (10 to 16)^a^
Chen et al. *Foot Ankle Int.* 2020. [34]	3	26	5; 21	45.9 (17 to 77)	54 (30 to 95)^a^
Cho et al. *Foot Ankle Int.* 2020. [35]	3	63	39; 24	SF 37.9 (18 to 65); SB 40.9 (20 to 69)	16 (12 to 26)^a^
Thomas et al. *Foot Ankle Spec*. 2020. [36]	4	100	50; 50	Male 34.3 (19 to 76); Female 34.5 (19 to 69)	NR
Arzac Ulla I. *Foot Ankle Surg.* 2021. [37]	4	14	10; 4	32 (NR)	24^b^
Chen et al. *Injury*. 2021. [38]	3	32	23; 9	ORIF 42.8 (36.2 to 49.4); PRIF 36.4 (28.8 to 44.0);	43 (35.6 to 50.4)^a^
Eceviz et al. *J Invest Surg.* 2021. [39]	3	62	44; 18	38 (18 to 68)	57 (24 to 155)^a^
Garríguez-Pérez et al. *Foot Ankle Int.* 2021. [40]	4	42	15; 27	49 (NR)	51.6 (12—96)^a^
Mosca et al. *Injury.* 2021. [41]	4	15	8; 7	48.2 (26 to 68)	45.6 (12 to 72)^a^
So et al. *Foot Ankle Spec.* 2021. [42]	3	196	85; 111	ORIF 35.8 (NR); PA 48.6 (NR)	ORIF 15.3 (18.9); PA 20.4 (28.3)^a^
De Bruijn et al. *Injury.* 2022. [43]	3	26	12; 10	42.6 (NR)	NR
Rikken et al. *Injury.* 2022. [44]	3	47	30; 17	32.6 (16 to 71)	NR

*Cx* Conservative treatment, *NR* Not reported, *ORIF* Open reduction and internal fixation, *PA* Primary arthrodesis, *PRIF* Percutaneous reduction and internal fixation, *SB* Suture button fixation, *SF* Screw fixation, *Sx* Surgical treatment

^a^Mean

^b^Median

^c^Final

**Fig. 1:**
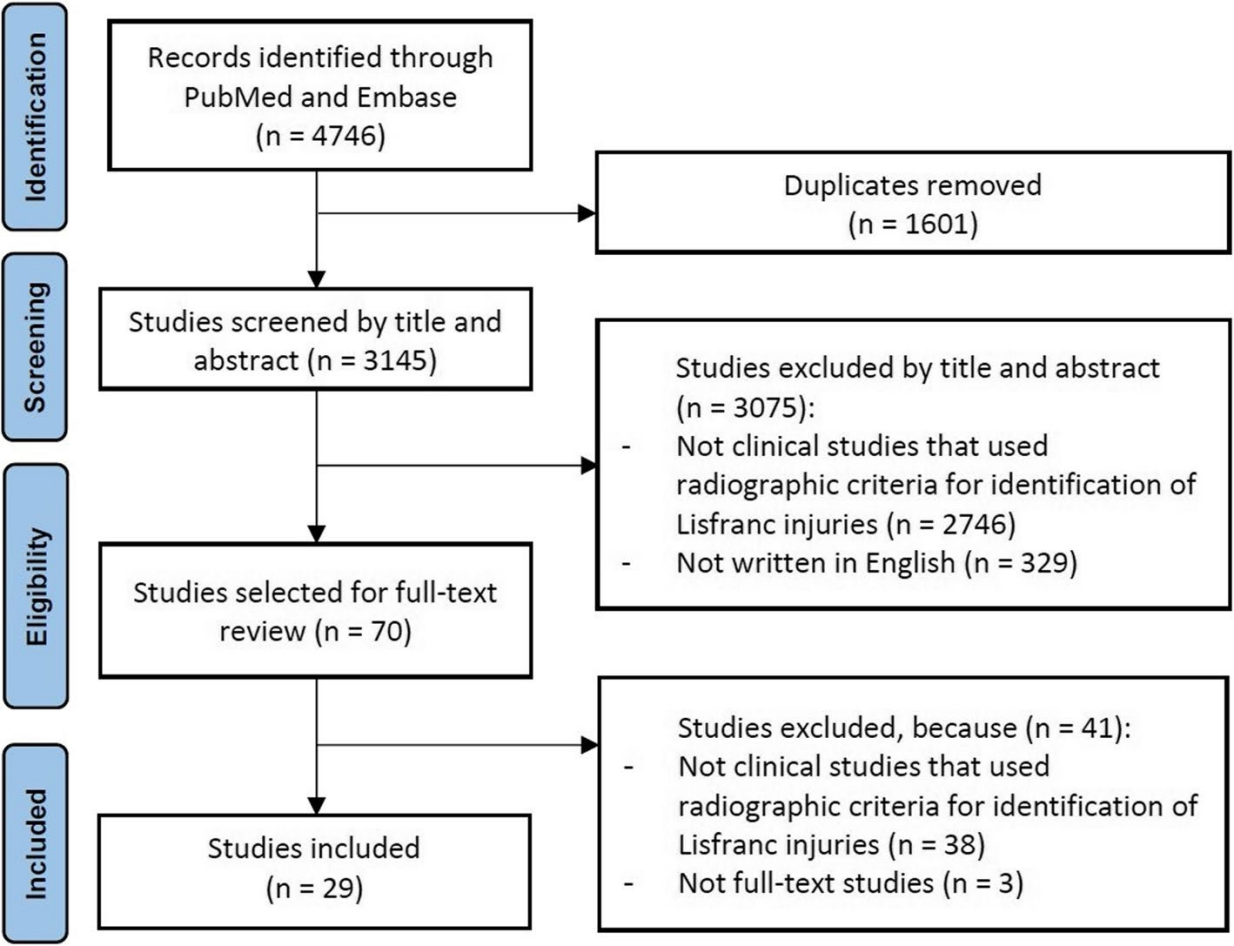
PRISMA flow diagram

**Fig. 2:**
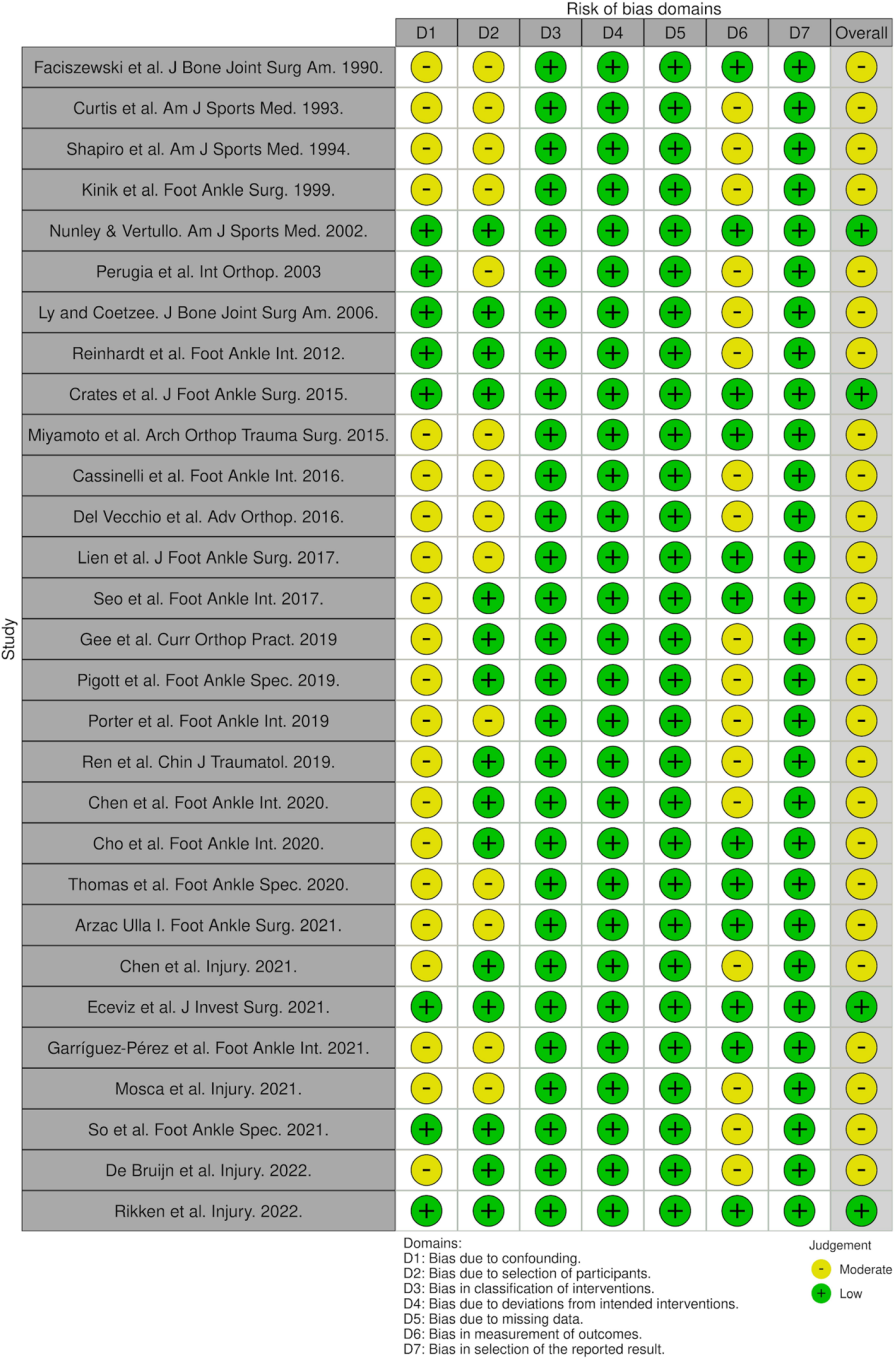
Breakdown of QoE assessment by the ROBINS-I Tool

A literature search based on the search strategy revealed 4746 studies for review (Fig. 1). There were 3075 studies excluded in the initial screening as they did not include Lisfranc injuries in their population or were not clinical cohort studies. There were 29 studies that met the eligibility criteria and therefore, were included. The included studies were published between 1990 and 2022 [16–44]. The mean LoE was 3.41 ± 0.68 (range, 1 to 4) according to the criteria by *The Journal of Bone and Joint Surgery.* The RoB ranged from “Low” to “Moderate” risk according to the ROBINS-I tool (Fig. 2). The overall recommendations according to the GRADE assessment ranged from “Very Low” to “High” (Table 2). All Lisfranc injuries were confirmed by radiographs as per eligibility criteria. This totalled 1115 Lisfranc injuries. This translated to 600 males, 470 females and 45 unreported genders. The mean age was 34.82 ± 8.63 (range, 19.40 to 49). The mean follow-up was 35.18 ± 15.02 (range, 12.30 to 58.40) months.

**Table 2:** GRADE assessment

Quality of evidence assessment							No. of patients diagnosed (% total injuries)	Overall quality of evidence	Importance
**No. of studies**	**Study design**	**Risk of bias**	**Inconsistency**	**Indirectness**	**Imprecision**	**Other considerations**			
**M1-M2 diastasis**									
18	Case series, Retrospective	Moderate	Serious^a^	Not serious	Not serious	None	468 (42.0)	Moderate	Important
**C2-M2 subluxation**									
11	Case series, Retrospective	Moderate	Not serious	Not serious	Not serious	None	562 (50.4)	High	Critical
**C1-M2 diastasis**									
8	Case series, Retrospective	Moderate	Serious^a^	Not serious	Not serious	None	377 (33.8)	Moderate	Moderate
**C3-M3 subluxation**									
7	Case series, Retrospective	Moderate	Not serious	Not serious	Not serious	None	372 (33.3)	Moderate	Moderate
**Longitudinal arch height**									
7	Case series, Retrospective	Moderate	Not serious	Not serious	Not serious	None	297 (26.6)	Low	Low
**Cuboid-M4 subluxation**									
7	Case series, Retrospective	Moderate	Not serious	Not serious	Not serious	None	392 (35.2)	Moderate	Moderate
**Talometatarsal angle**									
7	Case series, Retrospective	Moderate	Not serious	Not serious	Not serious	None	260 (23.3)	Low	Low
**Medial column malalignment (C1-M1)**									
6	Case series, Retrospective	Moderate	Not serious	Not serious	Not serious	None	397 (35.6)	Moderate	Moderate
**Cuneiform-metatarsal malalignment**									
6	Case series, Retrospective	Moderate	Not serious	Not serious	Not serious	None	345 (30.9)	Moderate	Moderate
**Fleck sign**									
6	Case series, Retrospective	Moderate	Serious^b^	Not serious	Not serious	None	110 (9.87)	Very low	Low
**Notch sign**									
1	Retrospective	Low	Not serious	Not serious	Not serious	None	36 (3.22)	Very low	Low

^a^Inconsistent measurement thresholds used across different studies for the same radiographic diagnostic criteria

^b^Within individual studies, some patients with Lisfranc injuries showed Fleck signs while others did not

### Characteristics (Fig. 3)

**Fig. 3:**
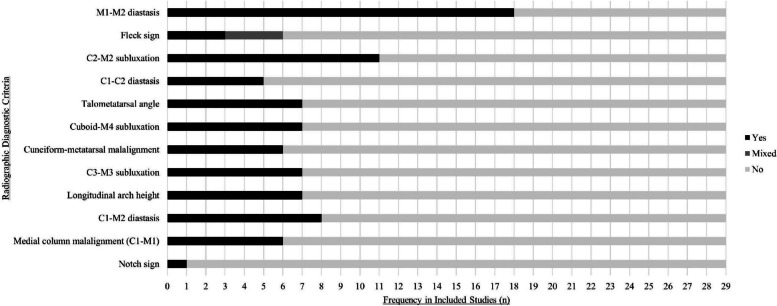
Frequency of radiographic diagnostic criteria for Lisfranc injuries

Weight-bearing radiographs were obtained in 12 studies [16, 19, 20, 25, 26, 28, 34–36, 39, 40, 44]. Two studies reported the use of (1) weight-bearing or non-weight-bearing radiographs [18, 43], and (2) weight-bearing and stress radiographs [33, 37]. One study used weight-bearing, non-weight-bearing, and stress radiographs [24]. The remaining 11 studies did not report the condition of their obtained radiographs [17, 21–23, 27, 30–32, 38, 41, 42]. Contralateral radiographs were fully obtained in 12 studies [16, 20, 24, 25, 28, 29, 35–37, 39, 40, 44]. One study was reported to have only obtained contralateral radiographs in four of nine of their patients [18], while another study obtained contralateral radiographs in three of eleven of their patients [19]. The remaining 15 studies did not report if contralateral radiographs were obtained for comparison [17, 21–23, 26, 27, 30–34, 38, 41–43]. The radiographic criteria reported in the 15 studies included (Table 3). In the anteroposterior view, the fleck sign, notch sign, medial column malalignment (C1-M1), C1-M2 diastasis, and M1-M2 diastasis. In the oblique view, C3-M3 subluxation, and cuboid-M4 subluxation. In the anteroposterior or oblique view, C1-C2 diastasis and C2-M2 subluxation. In the lateral view, cuneiform-metatarsal malalignment, longitudinal arch height, and talo-metatarsal angle.

**Table 3:** Radiographic diagnostic criteria for Lisfranc injuries

Study	Characteristics		Anteroposterior view					Oblique view		Anteroposterior or oblique view		Lateral view		
	Weightbearing condition(s)	Contralateral radiograph obtained	Fleck sign	Notch sign	Medial column malalignment (C1-M1)	C1-M2 diastasis	M1-M2 diastasis	C3-M3 subluxation	Cuboid-M4 subluxation	C1-C2 diastasis	C2-M2 subluxation	Cuneiform-metatarsal malalignment	Longitudinal arch height	Talo-metatarsal angle
Yes – the study used the radiographic criteria for diagnosis; No – the study explicitly reported that this radiographic criteria was not used for diagnosis; NR – the study did not comment on the radiographic criteria for diagnosis														
Faciszewski et al. *J Bone Joint Surg Am.* 1990. [16]	WB	Yes	NR	NR	NR	NR	2-5 mm	NR	NR	NR	NR	NR	NR	NR
Curtis et al. *Am J Sports Med.* 1993. [17]	NR	NR	Yes	NR	NR	NR	Yes	NR	NR	NR	NR	NR	NR	NR
Shapiro et al. *Am J Sports Med.* 1994. [18]	WB or NWB on a case basis	Yes, 4 (NWB); No, 5	NR	NR	NR	NR	Yes	NR	NR	NR	NR	NR	NR	NR
Kinik et al. *Foot Ankle Surg.* 1999. [19]	WB	Yes, 3; No 8	NR	NR	Yes	NR	> 2 mm	Yes	Yes	NR	NR	Yes	Yes	NR
Nunley and Vertullo. *Am J Sports Med.* 2002. [20]	WB	Yes	Yes	NR	NR	NR	> 1mm^a^	NR	NR	> 1mm^a^	Yes	NR	Yes	NR
Perugia et al. *Int Orthop.* 2003. [21]	NR	NR	NR	NR	NR	NR	Yes	Yes	Yes	NR	Yes	Yes	NR	Yes
Ly and Coetzee. *J Bone Joint Surg Am.* 2006. [22]	NR	NR	Yes	NR	NR	NR	NR	NR	NR	NR	NR	NR	NR	NR
Reinhardt et al. *Foot Ankle Int.* 2012. [23]	NR	NR	Yes, 12; No, 13	NR	NR	NR	Yes	NR	NR	Yes	NR	NR	NR	NR
Crates et al. *J Foot Ankle Surg.* 2015. [24]	WB, NWB, stress	Yes	NR	Yes	NR	NR	NR	NR	NR	NR	NR	Yes	Yes	Yes
Miyamoto et al. *Arch Orthop Trauma Surg.* 2015. [25]	WB	Yes	NR	NR	NR	NR	Yes	NR	NR	NR	NR	NR	NR	NR
Cassinelli et al. *Foot Ankle Int.* 2016. [26]	WB	NR	NR	NR	NR	NR	> 3 mm	NR	NR	NR	NR	NR	NR	NR
Del Vecchio et al. *Adv Orthop.* 2016. [27]	NR	NR	NR	NR	NR	NR	NR	Yes	NR	NR	Yes	NR	NR	Yes
Lien et al. *J Foot Ankle Surg.* 2017. [28]	WB	Yes	Yes, 4; No, 6	NR	NR	NR	Yes	NR	NR	NR	NR	NR	NR	NR
Seo et al. Foot Ankle Int. 2017. [29]	NWB	Yes	NR	NR	Yes	> 2 mm	NR	NR	NR	> 2 mm	Yes	NR	NR	NR
Gee et al. *Curr Orthop Pract.* 2019. [30]	NR	NR	NR	NR	NR	NR	Yes	NR	NR	NR	NR	NR	NR	NR
Pigott et al. Foot Ankle Spec. 2019. [31]	NR	NR	NR	NR	NR	NR	NR	Yes	Yes	NR	Yes	Yes	NR	NR
Porter et al. *Foot Ankle Int.* 2019. [32]	NR	NR	NR	NR	Yes	Yes	No	NR	NR	Yes	Yes	NR	NR	NR
Ren et al. *Chin J Traumatol.* 2019. [33]	WB, stress	NR	NR	NR	NR	NR	> 3 mm (WB)	NR	NR	NR	NR	NR	NR	NR
Chen et al. *Foot Ankle Int.* 2020. [34]	WB	NR	Yes, 19; No, 7	NR	NR	NR	Yes	NR	NR	NR	NR	NR	NR	NR
Cho et al. *Foot Ankle Int.* 2020. [35]	WB	Yes	NR	NR	NR	NR	> 2 mm	NR	NR	NR	NR	NR	NR	NR
Thomas et al. *Foot Ankle Spec*. 2020. [36]	WB	Yes	NR	NR	NR	Yes	NR	NR	NR	NR	NR	NR	Yes	No
Arzac Ulla I. *Foot Ankle Surg.* 2021. [37]	WB, stress	Yes	NR	NR	NR	> 2 mm	NR	NR	NR	NR	NR	NR	NR	NR
Chen et al. *Injury*. 2021. [38]	NR	NR	NR	NR	NR	NR	> 2 mm	NR	NR	NR	NR	NR	NR	NR
Eceviz et al. *J Invest Surg.* 2021. [39]	WB	Yes	NR	NR	NR	NR	NR	NR	NR	NR	NR	NR	Yes	Yes
Garríguez-Pérez et al. *Foot Ankle Int.* 2021. [40]	WB	Yes	NR	NR	Yes	3-5 mm	> 2mm^a^	NR	NR	NR	Yes	NR	NR	Yes
Mosca et al. *Injury.* 2021. [41]	NR	NR	NR	NR	Yes	> 2 mm	No	NR	NR	NR	NR	Yes	NR	NR
So et al. *Foot Ankle Spec.* 2021. [42]	NR	NR	NR	NR	Yes	NR	NR	Yes	Yes	NR	Yes	Yes	NR	NR
De Bruijn et al. *Injury.* 2022. [43]	WB, NWB	NR	NR	NR	NR	Yes	Yes	Yes	Yes	Yes	Yes	NR	Yes	Yes
Rikken et al. *Injury.* 2022. [44]	WB	Yes	NR	NR	NR	Yes	Yes	Yes	Yes	NR	Yes	NR	Yes	Yes

*Cuboid-M4* Cuboid to 4^th^ metatarsal, *C1-C2* 1^st^ cuneiform to 2^nd^ cuneiform, *C1-M1* 1^st^ cuneiform to 1^st^ metatarsal, *C1-M2* 1^st^ cuneiform to 2^nd^ metatarsal, *C2-M2* 2^nd^ cuneiform to 2^nd^ metatarsal, *C3-M3* 3^rd^ cuneiform to 3^rd^ metatarsal, *M1-M2* 1^st^ metatarsal to 2^nd^ metatarsal, *NR* Not reported, *NWB* Non-weight-bearing, *WB* Weight-bearing

^a^Compared to contralateral radiographs

#### Anteroposterior view

M1-M2 diastasis was the most common radiographic diagnostic criteria in the anteroposterior view, as was employed in 18 studies [16–21, 23, 25, 26, 28, 30, 33–35, 38, 40, 43, 44]. Specific distances for M1-M2 distance were reported in eight studies and employed at > 1 mm [22], > 2 mm [19, 35, 38, 40], > 3 mm [26, 33], or 2-5 mm [16]. 1^st^ cuneiform to 2^nd^ metatarsal diastasis was the second most common diagnostic criteria in the anteroposterior view and was employed in eight studies [29, 32, 36, 37, 40, 41, 43, 44]. Medial column alignment was reported in six studies [19, 29, 32, 40–42]. The fleck sign was fully employed in three studies [17, 20, 22] but mixed in three more other studies [23, 28, 34], and the notch sign was employed in one study [24].

#### Oblique view

3^rd^ cuneiform to 3rd metatarsal [19, 21, 27, 31, 42–44] and cuboid to 4^th^ metatarsal subluxation [19, 21, 23, 31, 42–44] were both the radiographic diagnostic criteria observed in the oblique view and employed in seven studies each.

#### Anteroposterior or oblique view

1^st^ cuneiform to 2^nd^ cuneiform diastasis and 2^nd^ cuneiform to 2^nd^ metatarsal subluxation were both the radiographic diagnostic criteria observed in the anteroposterior or oblique view. 2^nd^ cuneiform to 2^nd^ metatarsal subluxation was employed in 11 studies [19–21, 27, 29, 31, 32, 40, 42–44], whereas 1^st^ cuneiform to 2^nd^ cuneiform diastasis was employed in five studies [20, 23, 29, 32, 43].

#### Lateral view

The talometatarsal angle [21, 24, 27, 39, 40, 43, 44] and longitudinal arch height [19, 20, 24, 36, 39, 43, 44] were the most common radiographic diagnostic criteria in the lateral view, as employed in seven studies each. Cuneiform-metatarsal malalignment was employed in six studies [19, 21, 24, 31, 41, 42].

## Discussion

The heterogeneous diagnostic criteria of many disorders remain prominent across medicine [11, 45–47], and the radiographic diagnostic criteria of Lisfranc injuries are no exception [11]. Radiographs are a key first-line diagnostic tool for Lisfranc injuries [48]. However, in patients with normal radiographs but with continued suspicion of Lisfranc injuries, further advanced imaging by CT or MRI is suggested [49]. This systematic review reinforced that the radiographic diagnostic criteria for Lisfranc injuries have been heterogeneous. There are currently no clear guidelines or consensus on the use of radiographic criteria for the diagnosis of Lisfranc injuries. This becomes problematic because varying pathological characteristics can be indicative of varying severities that may then be associated with poorer prognosis. Potential reasons for heterogeneity may stem from the complex anatomy surrounding Lisfranc injuries. From the included studies, it can be observed that diastasis between several bony landmarks can be used in identifying Lisfranc injuries. While this review concluded that the most commonly observed diastasis is at M1-M2 and C1-C2, there are no studies correlating radiological findings and clinical presentation of Lisfranc injuries (i.e. patient symptoms, functional scores). Hence, it is difficult to ascertain which diastasis may bear the most clinical significance. In addition, differences in institutional practices and protocols for diagnosing Lisfranc injuries may also contribute to heterogeneity of current diagnostic criteria. It is important to consider these potential reasons for heterogeneity when discussing and standardising radiographic diagnostic criteria for Lisfranc injuries to improve consistency of diagnoses. In a retrospective case series of 51 patients that examined pre-surgical non-weight-bearing radiographs to intra-surgical stability, it was revealed that 1^st^ cuneiform to 2^nd^ metatarsal avulsion (fleck sign on radiographs) and 1^st^ cuneiform to 2^nd^ cuneiform diastasis were strong pre-surgical radiographic predictors of instability [29]. Therefore, the generalised outcomes following Lisfranc injuries must be currently interpreted with caution, with homogenous radiographic diagnostic criteria urged to be established.

The fleck sign is another radiographic sign that has been recognised to be an indicator of primarily ligamentous injuries in some studies [22, 23] but not all studies [17, 20, 28, 34]. There appeared to be similar outcomes across the included studies with the fleck sign compared to those without the fleck sign on radiographs for Lisfranc injuries. Many surgeons have agreed that purely ligamentous injuries may require a longer healing time than their bony counterparts [26]. However, this was not observed for Lisfranc injuries based on the fleck sign recorded on radiographs in the current systematic review. A plausible reason may be due to the heterogeneous radiographic diagnostic criteria observed, and its subsequent potential to yield a possible ambiguous outcome. This may have also been confounded by only three studies having reported clinical outcomes with the Fleck sign [17, 20, 22].

Further to the inconsistent radiographic diagnostic criteria observed, there were studies excluded during the literature search because these studies reported radiographic indications for surgery rather than diagnostic criteria [50–52]. The decision to exclude these studies was based on the fact that indication for surgery is inherently different from diagnosis criteria. This circumstance calls into question the difference, if any, between the radiographic diagnostic criteria and radiographic indication for surgery for Lisfranc injuries. This scenario importantly emphasises the inconsistency present for not just radiographic diagnostic criteria but also the possibility of the radiographic indication for surgery and even the radiographic alignment criteria following the treatment for Lisfranc injuries. These inconsistencies further question the consensual understanding of Lisfranc injuries as a whole in the current literature. The establishment of a consensus must be rapidly made for the best clinical practice.

Based on the radiographic diagnostic criteria findings of this systematic review, the following proposed homogenous radiographic diagnostic criteria for Lisfranc injuries is 1^st^ metatarsal to 2^nd^ metatarsal diastasis of ≥ 2 mm on anteroposterior view or 2^nd^ cuneiform to 2^nd^ metatarsal subluxation on anteroposterior or oblique views. As observed in this systematic review, there have been varying degrees of 1^st^ metatarsal to 2^nd^ metatarsal diastasis that conferred a Lisfranc injury diagnosis. Still, it remains difficult to ascertain the cut-off point (the minimum value observed per this systematic review was 1 mm). Further studies are warranted to ascertain this, but what can be safely noted is that the 1^st^ metatarsal to 2^nd^ metatarsal diastasis of ≥ 2 mm was by far the most common radiographic diagnostic criteria. The next most common radiographic criterion was 2^nd^ cuneiform to 2^nd^ metatarsal subluxation, although no cut-off values were reported among the included studies. Theoretically, these radiographic criteria are sound indicators of the separation of the metatarsi from the tarsus as per the characteristics of a Lisfranc injury [1]. Further radiographic views such as oblique radiographs may support diagnosis of Lisfranc injuries if 1^st^ metatarsal to 2^nd^ metatarsal diastasis or 2^nd^ cuneiform to 2^nd^ metatarsal subluxation on anteroposterior views are equivocal. It is also proposed that the presence of a fleck sign is not to be necessarily included as a radiographic diagnostic criterion. Despite the fleck sign being recognised as a representation of primarily ligamentous injuries in some studies, there appeared to be no apparent association between the fleck sign on radiographs and the clinical outcomes of Lisfranc injuries. However, this may be confounded by fewer studies having reported clinical outcomes with than without the Fleck sign (three versus 26 studies, respectively). These propositions would ideally be in bilateral weight-bearing radiographs, although it is foreseeable that this might not always be possible in the acute setting. It is also ideal to have metatarsi alignment to their respective tarsi. Still, it is proposed that this is not to be made necessary and hence have not been included as part of the radiographic diagnostic criteria for Lisfranc injuries. Previous studies have demonstrated that radiographic measurements of tarsometatarsal alignment have limited ability to detect Lisfranc injuries because of the innate nature of tarsometatarsal anatomy that there is a normal step-off of the metatarsi edge compared to their respective tarsi [9, 11].

The current literature has also debated the superiority of radiographs compared to CT and MRI [9, 48, 53]. Radiographs are non-invasive, inexpensive, and rapidly available and therefore, are theoretically sound to be conferred as first-line. Some benefits of image acquisition by CT include greater detailed imaging for operative planning. CT has also been demonstrated to be best at detecting small bony displacements that may be otherwise missed in non-weight-bearing radiographs [54]. However, CT scans are more expensive compared to plain radiographs and any advantages that CT images provide must ultimately justify the increased cost. Kennelly et al. did suggest that these benefits are limited and may not impact management of Lisfranc injuries. In fact, only 12% of CT scans subsequently detected Lisfranc injuries after initial weight bearing radiographs were negative [48]. MRI imaging is known to be an excellent tool to assess soft tissue structures including the Lisfranc ligament, which is the interosseous ligament between the 1st cuneiform and 2nd metatarsal [55]. Kitsukawa et al. suggested that due to the oblique course of the Lisfranc ligament with respect to the anatomical body axis, 3-dimensional MRI is advantageous to assess Lisfranc injuries. In their study, the authors concluded that MRI identified Lisfranc ligament (interosseous C1-C2 ligament) injuries corresponded with intraoperative findings in all included patients [55]. This suggests that MRI provides excellent diagnostic accuracy for Lisfranc injuries. However, MRI does not appear suitable for first-line diagnosis due to its inherent nature to require a noteworthy amount of time for image acquisition and reduced ease of access compared to plain radiographs. In addition, using MRI as a first-line imaging modality may pose the risk of overdiagnosis in lower grade injuries as suggested by Macmohan et al. [56]. Therefore, this reiterates the importance of concrete radiographic diagnostic criteria to be rapidly achieved. Radiographic criteria between conventional radiographs and CT share some familiar imagery and therefore, can somewhat be cross-shared to have common diagnostic criteria between the two platforms. Noticeably, the fleck sign is visible on both radiographs and CT. In patients with normal radiographs but with continued suspicion of Lisfranc injuries, further advanced imaging by CT or MRI is suggested [49].

The strength of this study was that a comprehensive search strategy was employed to encompass all varying severities of Lisfranc injuries. However, there were several limitations to this study. These limitations can be divided into limitations of the systematic review and the limitations of the included studies. The limitations of the systematic review were that the eligibility was only limited to full-text studies written in English, which may have predisposed selection bias. Reviews have also been noted to summarise and aggregate data and may have comprised intrinsic bias [57]. In addition, the level of heterogeneity was not objectively quantified as the data obtained from included studies was inherently unsuited for meta-analysis. The limitations of the included studies were that inter-operator variability of the obtained radiographs for diagnosis in the included studies was inherently present. Factors beyond operator control may have also influenced the radiographs in the clinical setting. This may have included but was not limited to foot position, muscular tone, muscular relaxation, patient orientation and patient posture. The included studies did not report rates of missed diagnosis pertaining to each radiographic criterion. Hence the diagnostic accuracy of each radiographic sign could not be objectively evaluated, which limits clinical decision-making regarding which criteria to use in diagnosing Lisfranc injuries. The location of Lisfranc injuries (medial or lateral column) was not reported in the included studies. This limits the objective evaluation of the diagnostic accuracy of the proposed radiographic diagnostic criteria with respect to different classifications of Lisfranc injuries.

In conclusion, the radiographic diagnostic criteria of Lisfranc injuries were heterogeneous. The proposition for homogenous radiographic diagnostic criteria is that the following features must be observed for the diagnosis of Lisfranc injuries: 1^st^ metatarsal to 2^nd^ metatarsal diastasis on anteroposterior view or 2^nd^ cuneiform to 2^nd^ metatarsal subluxation on anteroposterior or oblique views. Further advanced imaging by CT or MRI may be required in patients with normal radiographs but with continued suspicion for Lisfranc injuries. Future studies are warranted to investigate the proposed radiographic diagnostic criteria and their association with clinical outcomes for Lisfranc injuries. Notably, if any radiographic diagnostic criteria can indicate severity or associated with poorer prognosis.

## Data Availability

Available on request to email Dr Dexter Seow at dexterseow@rcsi.ie.
